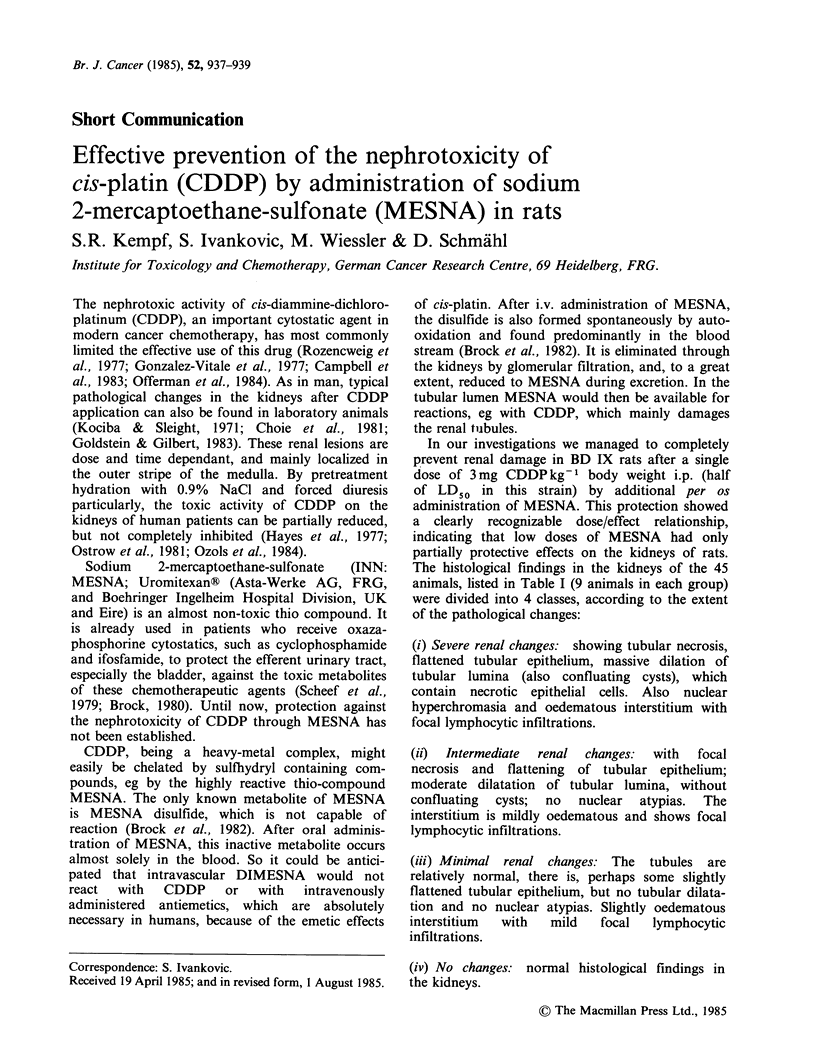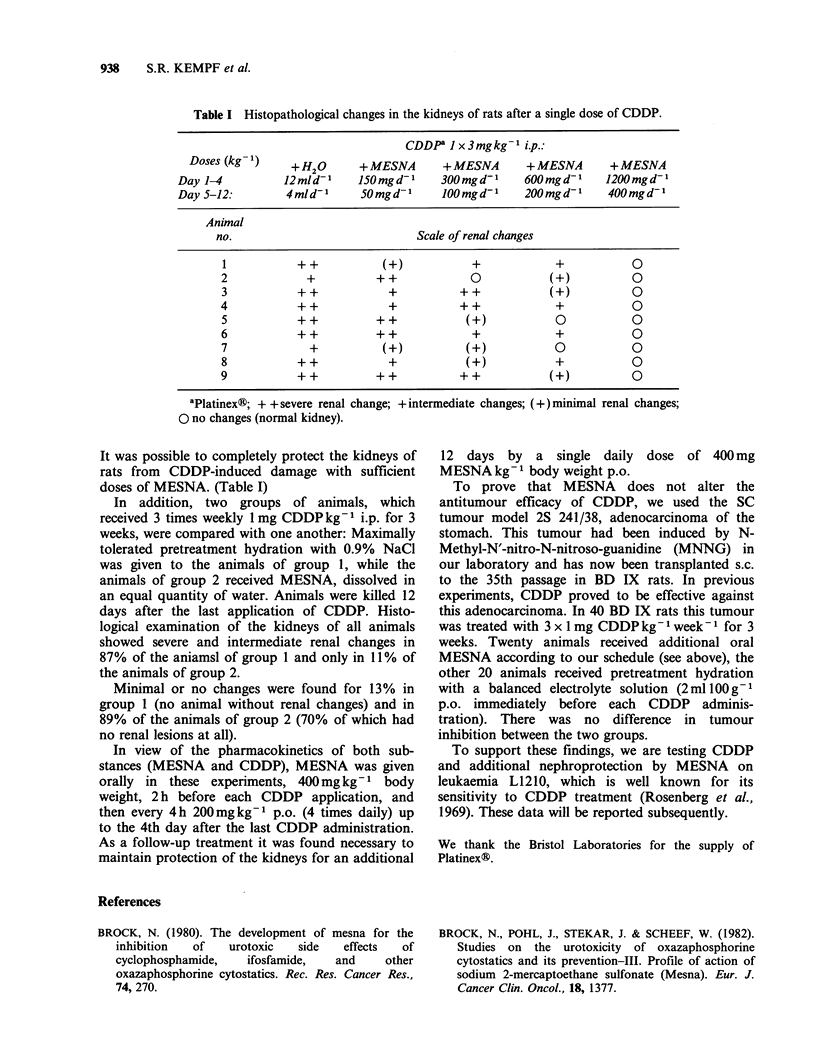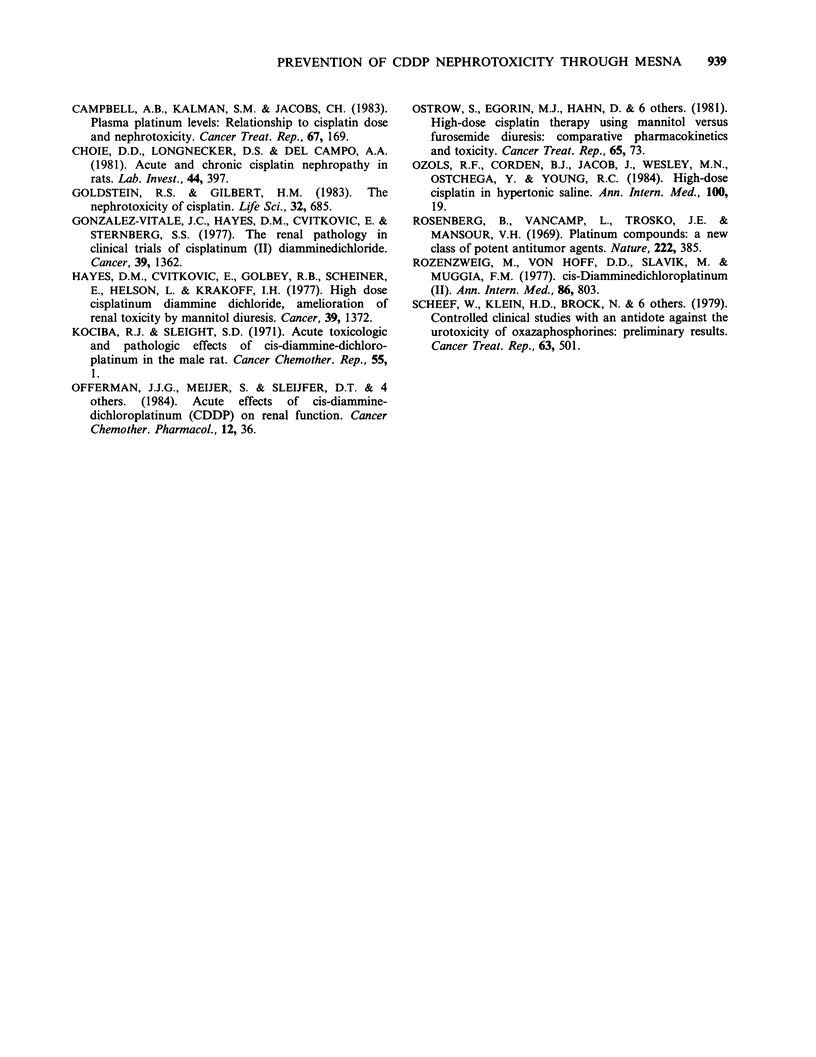# Effective prevention of the nephrotoxicity of cis-platin (CDDP) by administration of sodium 2-mercaptoethane-sulfonate (MESNA) in rats.

**DOI:** 10.1038/bjc.1985.280

**Published:** 1985-12

**Authors:** S. R. Kempf, S. Ivankovic, M. Wiessler, D. Schmähl


*Br. J. Cancer* (1985), **52**, 937–939

### Short Communication

### Effective prevention of the nephrotoxicity of *cis*-platin (CDDP) by administration of sodium 2-mercaptoethane-sulfonate (MESNA) in rats

S.R. Kempf, S. Ivankovic, M. Wiessler & D. Schmähl

*Institute for Toxicology and Chemotherapy, German Cancer Research Centre, 69 Heidelberg, FRG.*

The nephrotoxic activity of *cis*-diammine-dichloroplatinum (CDDP), an important cytostatic agent in modern cancer chemotherapy, has most commonly limited the effective use of this drug (Rozencweig *et al.*, 1977; Gonzalez-Vitale *et al.*, 1977; Campbell *et al.*, 1983; Offerman *et al.*, 1984). As in man, typical pathological changes in the kidneys after CDDP application can also be found in laboratory animals (Kociba & Sleight, 1971; Choie *et al.*, 1981; Goldstein & Gilbert, 1983). These renal lesions are dose and time dependant, and mainly localized in the outer stripe of the medulla. By pretreatment hydration with 0.9% NaCl and forced diuresis particularly, the toxic activity of CDDP on the kidneys of human patients can be partially reduced, but not completely inhibited (Hayes *et al.*, 1977; Ostrow *et al.*, 1981; Ozols *et al.*, 1984).

Sodium 2-mercaptoethane-sulfonate (INN: MESNA; Uromitexan® (Asta-Werke AG, FRG, and Boehringer Ingelheim Hospital Division, UK and Eire) is an almost non-toxic thio compound. It is already used in patients who receive oxazaphosphorine cytostatics, such as cyclophosphamide and ifosfamide, to protect the efferent urinary tract, especially the bladder, against the toxic metabolites of these chemotherapeutic agents (Scheef *et al.*, 1979; Brock, 1980). Until now, protection against the nephrotoxicity of CDDP through MESNA has not been established.

CDDP, being a heavy-metal complex, might easily be chelated by sulfhydryl containing compounds, eg by the highly reactive thio-compound MESNA. The only known metabolite of MESNA is MESNA disulfide, which is not capable of reaction (Brock *et al.*, 1982). After oral administration of MESNA, this inactive metabolite occurs almost solely in the blood. So it could be anticipated that intravascular DIMESNA would not react with CDDP or with intravenously administered antiemetics, which are absolutely necessary in humans, because of the emetic effects of *cis*-platin. After i.v. administration of MESNA, the disulfide is also formed spontaneously by autooxidation and found predominantly in the blood stream (Brock *et al.*, 1982). It is eliminated through the kidneys by glomerular filtration, and, to a great extent, reduced to MESNA during excretion. In the tubular lumen MESNA would then be available for reactions, eg with CDDP, which mainly damages the renal tubules.

In our investigations we managed to completely prevent renal damage in BD IX rats after a single dose of 3 mg CDDP $kg^{-1}$ body weight i.p. (half of $LD_{50}$ in this strain) by additional *per os* administration of MESNA. This protection showed a clearly recognizable dose/effect relationship, indicating that low doses of MESNA had only partially protective effects on the kidneys of rats. The histological findings in the kidneys of the 45 animals, listed in Table I (9 animals in each group) were divided into 4 classes, according to the extent of the pathological changes:

(*i*) *Severe renal changes:* showing tubular necrosis, flattened tubular epithelium, massive dilation of tubular lumina (also confluating cysts), which contain necrotic epithelial cells. Also nuclear hyperchromasia and oedematous interstitium with focal lymphocytic infiltrations.

(*ii*) *Intermediate renal changes:* with focal necrosis and flattening of tubular epithelium; moderate dilatation of tubular lumina, without confluating cysts; no nuclear atypias. The interstitium is mildly oedematous and shows focal lymphocytic infiltrations.

(*iii*) *Minimal renal changes:* The tubules are relatively normal, there is, perhaps some slightly flattened tubular epithelium, but no tubular dilatation and no nuclear atypias. Slightly oedematous interstitium with mild focal lymphocytic infiltrations.

(*iv*) *No changes:* normal histological findings in the kidneys.

Correspondence: S. Ivankovic.
Received 19 April 1985; and in revised form, 1 August 1985.

© The Macmillan Press Ltd., 1985

**Table I** Histopathological changes in the kidneys of rats after a single dose of CDDP.

| | *CDDP[a] 1 × 3 mg kg$^{-1}$ i.p.:* | | | | |
|---|---|---|---|---|---|
| *Doses (kg$^{-1}$)* | *+$H_2O$* | *+MESNA* | *+MESNA* | *+MESNA* | *+MESNA* |
| *Day 1–4* | *12 ml d$^{-1}$* | *150 mg d$^{-1}$* | *300 mg d$^{-1}$* | *600 mg d$^{-1}$* | *1200 mg d$^{-1}$* |
| *Day 5–12:* | *4 ml d$^{-1}$* | *50 mg d$^{-1}$* | *100 mg d$^{-1}$* | *200 mg d$^{-1}$* | *400 mg d$^{-1}$* |
| *Animal no.* | *Scale of renal changes* | | | | |
| 1 | ++ | (+) | + | + | ○ |
| 2 | + | ++ | ○ | (+) | ○ |
| 3 | ++ | + | ++ | (+) | ○ |
| 4 | ++ | + | ++ | + | ○ |
| 5 | ++ | ++ | (+) | ○ | ○ |
| 6 | ++ | ++ | + | + | ○ |
| 7 | + | (+) | (+) | ○ | ○ |
| 8 | ++ | + | (+) | + | ○ |
| 9 | ++ | ++ | ++ | (+) | ○ |

[a]Platinex®; ++ severe renal change; + intermediate changes; (+) minimal renal changes; ○ no changes (normal kidney).

It was possible to completely protect the kidneys of rats from CDDP-induced damage with sufficient doses of MESNA. (Table I)

In addition, two groups of animals, which received 3 times weekly 1 mg CDDP kg$^{-1}$ i.p. for 3 weeks, were compared with one another: Maximally tolerated pretreatment hydration with 0.9% NaCl was given to the animals of group 1, while the animals of group 2 received MESNA, dissolved in an equal quantity of water. Animals were killed 12 days after the last application of CDDP. Histological examination of the kidneys of all animals showed severe and intermediate renal changes in 87% of the aniamsl of group 1 and only in 11% of the animals of group 2.

Minimal or no changes were found for 13% in group 1 (no animal without renal changes) and in 89% of the animals of group 2 (70% of which had no renal lesions at all).

In view of the pharmacokinetics of both substances (MESNA and CDDP), MESNA was given orally in these experiments, 400 mg kg$^{-1}$ body weight, 2 h before each CDDP application, and then every 4 h 200 mg kg$^{-1}$ p.o. (4 times daily) up to the 4th day after the last CDDP administration. As a follow-up treatment it was found necessary to maintain protection of the kidneys for an additional 12 days by a single daily dose of 400 mg MESNA kg$^{-1}$ body weight p.o.

To prove that MESNA does not alter the antitumour efficacy of CDDP, we used the SC tumour model 2S 241/38, adenocarcinoma of the stomach. This tumour had been induced by N-Methyl-N′-nitro-N-nitroso-guanidine (MNNG) in our laboratory and has now been transplanted s.c. to the 35th passage in BD IX rats. In previous experiments, CDDP proved to be effective against this adenocarcinoma. In 40 BD IX rats this tumour was treated with 3 × 1 mg CDDP kg$^{-1}$ week$^{-1}$ for 3 weeks. Twenty animals received additional oral MESNA according to our schedule (see above), the other 20 animals received pretreatment hydration with a balanced electrolyte solution (2 ml 100 g$^{-1}$ p.o. immediately before each CDDP administration). There was no difference in tumour inhibition between the two groups.

To support these findings, we are testing CDDP and additional nephroprotection by MESNA on leukaemia L1210, which is well known for its sensitivity to CDDP treatment (Rosenberg *et al.*, 1969). These data will be reported subsequently.

We thank the Bristol Laboratories for the supply of Platinex®.

### References

BROCK, N. (1980). The development of mesna for the inhibition of urotoxic side effects of cyclophosphamide, ifosfamide, and other oxazaphosphorine cytostatics. *Rec. Res. Cancer Res.*, **74,** 270.

BROCK, N., POHL, J., STEKAR, J. & SCHEEF, W. (1982). Studies on the urotoxicity of oxazaphosphorine cytostatics and its prevention–III. Profile of action of sodium 2-mercaptoethane sulfonate (Mesna). *Eur. J. Cancer Clin. Oncol.*, **18,** 1377.

CAMPBELL, A.B., KALMAN, S.M. & JACOBS, CH. (1983). Plasma platinum levels: Relationship to cisplatin dose and nephrotoxicity. *Cancer Treat. Rep.*, **67**, 169.

CHOIE, D.D., LONGNECKER, D.S. & DEL CAMPO, A.A. (1981). Acute and chronic cisplatin nephropathy in rats. *Lab. Invest.*, **44**, 397.

GOLDSTEIN, R.S. & GILBERT, H.M. (1983). The nephrotoxicity of cisplatin. *Life Sci.*, **32**, 685.

GONZALEZ-VITALE, J.C., HAYES, D.M., CVITKOVIC, E. & STERNBERG, S.S. (1977). The renal pathology in clinical trials of cisplatinum (II) diamminedichloride. *Cancer*, **39**, 1362.

HAYES, D.M., CVITKOVIC, E., GOLBEY, R.B., SCHEINER, E., HELSON, L. & KRAKOFF, I.H. (1977). High dose cisplatinum diammine dichloride, amelioration of renal toxicity by mannitol diuresis. *Cancer*, **39**, 1372.

KOCIBA, R.J. & SLEIGHT, S.D. (1971). Acute toxicologic and pathologic effects of cis-diammine-dichloroplatinum in the male rat. *Cancer Chemother. Rep.*, **55**, 1.

OFFERMAN, J.J.G., MEIJER, S. & SLEIJFER, D.T. & 4 others. (1984). Acute effects of cis-diammine-dichloroplatinum (CDDP) on renal function. *Cancer Chemother. Pharmacol.*, **12**, 36.

OSTROW, S., EGORIN, M.J., HAHN, D. & 6 others. (1981). High-dose cisplatin therapy using mannitol versus furosemide diuresis: comparative pharmacokinetics and toxicity. *Cancer Treat. Rep.*, **65**, 73.

OZOLS, R.F., CORDEN, B.J., JACOB, J., WESLEY, M.N., OSTCHEGA, Y. & YOUNG, R.C. (1984). High-dose cisplatin in hypertonic saline. *Ann. Intern. Med.*, **100**, 19.

ROSENBERG, B., VANCAMP, L., TROSKO, J.E. & MANSOUR, V.H. (1969). Platinum compounds: a new class of potent antitumor agents. *Nature*, **222**, 385.

ROZENZWEIG, M., VON HOFF, D.D., SLAVIK, M. & MUGGIA, F.M. (1977). cis-Diamminedichloroplatinum (II). *Ann. Intern. Med.*, **86**, 803.

SCHEEF, W., KLEIN, H.D., BROCK, N. & 6 others. (1979). Controlled clinical studies with an antidote against the urotoxicity of oxazaphosphorines: preliminary results. *Cancer Treat. Rep.*, **63**, 501.